# An Overview of Food Patterns and Diet Quality in Qatar: Findings from the National Household Income Expenditure Survey

**DOI:** 10.7759/cureus.1249

**Published:** 2017-05-15

**Authors:** Mohammed Al-Thani, Al-Anoud Al-Thani, Nasser Al-Mahdi, Hefzi Al-Kareem, Darine Barakat, Walaa Al-Chetachi, Afaf Tawfik, Hammad Akram

**Affiliations:** 1 Ministry of Public Health, State of Qatar; 2 Ministry of Development Planning and Statistics; 3 National Nutrition Institute

**Keywords:** dietary patterns, household expenditure survey, nutrition, qatar

## Abstract

**Introduction:**

Availability of accurate data pertaining to a population’s dietary patterns and associated health outcomes is critical for proper development and implementation of related policies. This article is a first attempt to share the food patterns, amounts and diet quality among households (HH) in Qatar.

**Methods:**

Data from the 2012-2013 Qatar National Household Income and Expenditure Survey (HIES) was used. This cross-sectional survey included 3723 HH (1826 Qatari HH and 1897 non-Qatari HH). Dietary data on monthly amounts food items available at HH according to the nationality was used. The food items were expressed in terms of grams per capita per day and aggregated into groups to examine the food patterns, energy, and adequacy.

**Results:**

The overall average amount of purchased food at HH in Qatar was 1885 g/capita/day. Qatari HH purchased more food (2118 g/capita/day) versus non-Qataris (1373 g/capita/day); however, the percentages of the amounts purchased by food types were similar among both nationalities. Average daily energy (kcal) per capita was almost double among Qatari HH (4275 kcal) vs. non-Qatari HH (2424 kcal). The food items under subsidy program for Qatari citizens provided 1753 kcal/capita/day and accounted for 41% of total daily energy. Proteins (29.2), fats (39.2), sodium (3.3), and vitamin C (32.5) had higher than recommended levels of nutrient density (grams per 1000 kcal). Calcium (227), vitamin A (302.3), fiber (2.0), and carbohydrates (132.6) had lower than recommended levels of nutrient energy density (g/1000 kcal).

**Conclusions:**

The study predicts unhealthy dietary habits among HH in Qatar and provides useful information for policy makers and healthcare community.

## Introduction

Availability of accurate data pertaining to a population’s dietary patterns and associated health outcomes is critical for proper development and implementation of related policies [1]. Food and nutrition policies then contribute positively to the health promotion initiatives in a country. The dietary patterns among populations could be assessed through using tools such as food balance sheets and the household (HH) surveys, which collect information on budget, expenditure, and income, or through specific individual-level dietary consumption studies [1]. While long-term planning for expansion of food availability is usually derived from food balance-sheet figures, the data from household expenditure surveys provide supplemental information about important food parameters such as diet quality, adequacy, and expenditure, etc. [2].

The household expenditure surveys provide valuable information pertaining to the various socio-economic indicators of a population. Most of the expenditure surveys are a part of larger public health initiatives and could provide information related to the income, expenditure, food consumption, and the consumption of other non-food items among households [1]. The data derived from these surveys can provide a reasonable understanding of food affordability, related behaviors, and, to some extent, the perception of healthy food habits among households [2].

The dietary habits of a community could play an important role in the various health outcomes such as cardiovascular disease, certain cancers, diabetes, and obesity [3]. Obesity is a major public health issue globally and also among Arab countries [4]. The State of Qatar, a country located in the Arab region with a population of over 2.4 million, has undergone rapid socio-economic development in the past few decades [5]. This progress and development have impacted the lifestyle and dietary habits in Qatar. Qatari residents are consuming more animal proteins, fats, and refined carbohydrates compared with the fiber-rich diets, which include consumption of items such as fruits, vegetables, and whole grains [6]. This is evident from the results of 2012 Qatar’s National STEPwise survey, according to which 91% of Qataris were consuming less than (recommended) five servings of fruit and/or vegetables per day [7]. Furthermore, along with the change in dietary habits, obesity prevalence was also found to be high (41.4%) among Qataris [7]. In Qatar, cardiovascular and metabolic diseases (including diabetes) are the leading causes of non-communicable disease-related deaths along with cancers and are considered as priority conditions, as they can possibly be controlled by diet and/or behavioral modification [8].

The present article provides the findings from the food data collected during the 2012-2013 Qatar Household Income Expenditure Survey (HIES). This article is the first attempt to share important food-related parameters in Qatar and provide details of food patterns and quality of diet based on nutrient density recommendation. The results may assist policy makers and program implementers to monitor and evaluate existing strategies such as Qatar Dietary Guidelines initiative and to design new evidence-based programs.

## Materials and methods

Secondary analysis was carried out using data from the HIES. The HIES was planned and conducted by the Ministry of Development Planning and Statistics (MDPS) between September 2012 and September 2013. The survey included 3723 HHs (1826 Qatari HHs and 1897 non-Qatari HHs). Data was collected as per accordance to the MDPS protocol and methodology for HIES [9]. A two-stage sample design was used by selecting primary sampling units (PSU) (constructed by combining census blocks) in first stage and sampling of households (Qatari and non-Qatari) in second stage. This was carried out on the basis of 2010 census population, housing, and establishments in the country [9].

The dietary data on food included a total of 327 food items available at HH (purchased & home produced), representing different food groups. Purchased food included the food obtained from the market and/or through food subsidy system (for Qatari HHs only). The data for home produced food items were only collected for Qatari HHs due to their housing types (houses with sufficient land to produce). The following food groups were included in the collecting data sheet: cereals and cereal products, meat and poultry, fish and sea foods, milk and dairy products, egg, oils and fats, fruit, vegetables, tubers and tuber products, legumes, nuts and seeds, salt and pickles, sugar and sweets, beverages and soft drinks. The group of cereals and cereal products included different types of rice, wheat and wheat products, different types of bread, macaroni, cakes, biscuits and oriental sweets. Meat and poultry group include different types of red meat (lamb, beef, and camel meat) either fresh or frozen, liver, organs, red meat processed products and poultry (fresh or frozen). The group of fish and seafoods included the most common types of fish and seafoods in the State of Qatar: fresh, frozen, and canned. Milk and dairy group include all the types of milk (fresh, condensed, dried powder, either full cream, half cream or skimmed milk) as well as yoghurt (full cream or half cream), processed and white cheese and other types of dairy products. Egg was reported in one group in data sheet. Different types of oils (sunflower, corn, palm, soybean, olive, coconut) besides butter, ghee, and margarine were included in oil and fat group. The group of fruit contained all fresh fruit as well as dried and canned fruit. The vegetables group included all fresh, frozen, and canned vegetables. The tubers group included potato, sweet potato, either fresh or frozen besides potato products (such as chips). Legume group included raw dried lentils, beans, peas, lupine, chickpea as well as their canned form. Nuts and seeds group contained peeled and unpeeled types of nuts. Salt and types of spices besides pickles and different types of chicken and beef stocks were included in this group. Free sugars (ration and non-ration) as well as kinds of jam, honey, molasses, sweets, gums, and chocolates were the food items included in sugar and sweets group. Tea, coffee, and cocoa powder were reported in one group. The group of beverages included soft carbonated drinks, fresh and canned vegetables and fruit juice as well as different types of water (treated drinking water, mineral, and carbonated water). The last group was the home made products or gifts (lamb, beef, poultry, fish and seafoods, milk and dairy products, egg, margarine, honey, dates, pickles, and different types of vegetables and fruit).

The amount of each food item was presented in kilograms for the household per month. As a first step, the amount was converted to grams, and then divided by 30 days then the resultant was divided by the average family size in order to be expressed as gram/day/capita according to the nationality of HHs categorized as Qatari and non-Qatari. The amounts of food items were aggregated according to nine food groups. Amounts and percentages of each food group were categorized by nationality and calculated using IBM SPSS version 16.0, Chicago, SPSS Inc.

After reviewing the Food Composition Tables (FCT) from Bahrain, Egypt, Ireland, and the United States of America, FCT of Egypt (National Nutrition Institute-2006) was used for analyzing food items to its nutrient content as it contained almost all the food items purchased by the surveyed HH [10]. Only one food item (Iranian bread) was analyzed using Bahraini FCT [11]. Both FCTs were appropriately selected and coincided with the data from Qatar survey. The daily amount of purchased food item per person was analyzed for its nutrient content using FCTs after taking consideration for non-edible portion or refuse in certain food items [10]. The analysis was performed for energy contents, macronutrients (proteins, fat, carbohydrates), fiber, and micro-nutrients (vitamins A & C; minerals iron, calcium, and sodium).

Nutritional requirements for the sample population (Qatari & non-Qatari and total sample) were estimated considering the structure by age and gender and the nutritional requirements set by the World Health Organization (WHO) [12]. The weighted daily per capita requirement for energy was estimated in terms of kcal, while protein requirement estimate (as reference protein) was adjusted according to the dietary pattern (gm/caput/day) [12-13]. Micronutrients requirements were calculated according to the criteria stated by Food and Agriculture Organization (FAO) and WHO [14]. Adequacy of macro- and micro-nutrients as well as energy was computed by comparing the energy and nutrient values of the food purchased with the estimated requirements and expressed as the percentage of requirements. Diet quality, which is essential to identify the balance of nutrients in the diet, was assessed through calculating energy pattern of the diet, and nutrient density per 1000 kcal energy [15-16].

Ethical procedures were followed throughout the study implementation, during data handling and analysis. Informed consent was carried out at the time of survey. Data was kept in secured, password protected computers.

## Results

**Figure 1:**
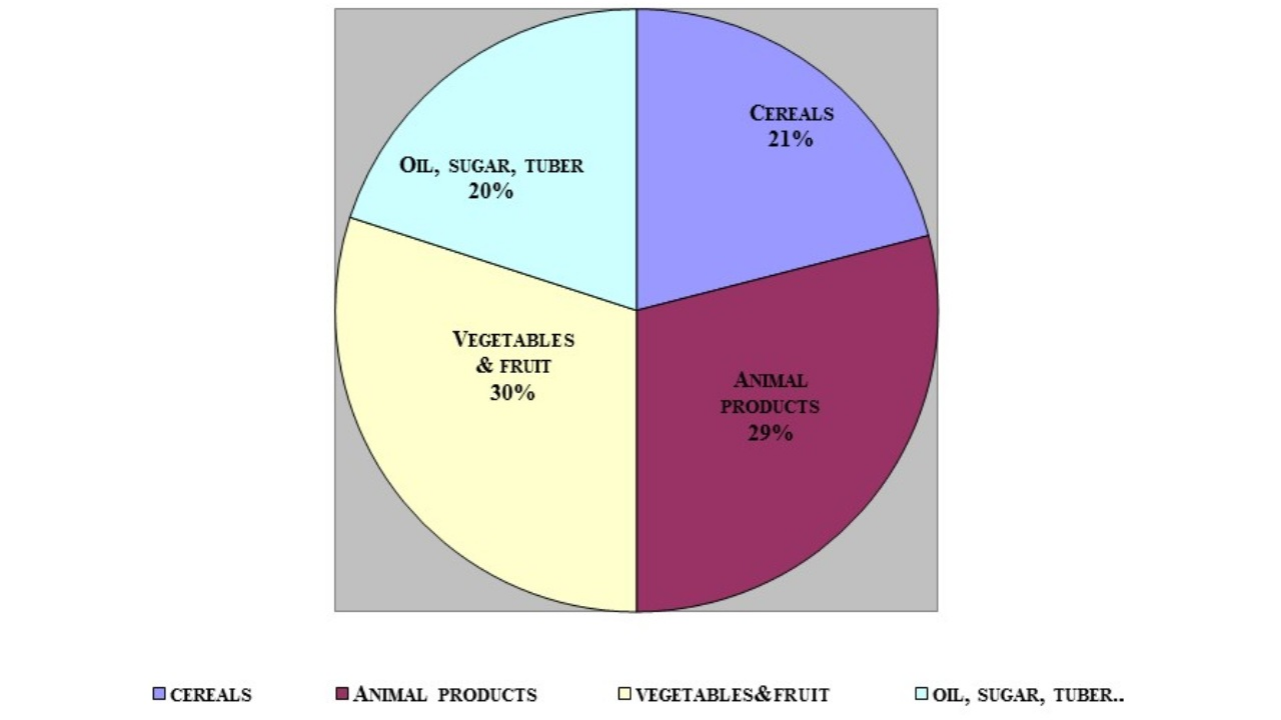
Food Basket Composition of Households (HHs) in Qatar (2012-2013), n = 3723 HHs

A total of 3723 HHs (1826 Qatari HHs and 1897 non-Qatari HHs) were surveyed and included in the analysis. The average family size for the total surveyed HHs was 5.49 higher for Qataris (8.65) versus non-Qataris (4.23). The overall average amount of purchased food at households in Qatar was 1885 g/capita/day (Table 1). The amounts differed widely by nationality, where Qatari HHs purchased more food (2118 g/capita/day) versus non-Qatari HH (1373 g/capita/day). Although the average daily per capita amounts (in grams) of all nine food groups were different by nationality, the percentages of the amounts purchased were similar by nationality and overall (Table 1). The vegetables and fruit group accounted for 30% of the food basket weight followed by all animal products (29%) and cereals (21%) (Table 1, Figure 1).

**Table 1:** Average daily purchased food per capita by nationality in the State of Qatar (2012-2013) HHs: Households

	Qatari (n = 1897 HHs)		Non-Qatari (n = 1826 HHs)		Total population (n = 3723 HHs)	
	Amount* (g)	(%)	Amount* (g)	(%)	Amount* (g)	(%)
Cereals	442	21	279	20	386	21
Meat, poultry & fish ^ǂ^	343	16	182	13	287	15
Milk, dairy products & eggs ^ǂ^	291	14	200	15	258	14
Tubers	63	3	40	3	55	3
Legumes & nuts	28	1	22	2	27	1
Vegetables & fruits	599	28	463	34	571	30
Oils & Fats	83	4	45	3	69	4
Sugar & sweets	153	7	55	4	126	7
Beverages	116	6	87	6	106	5
Total	2118	100	1373	100	1885	100
*Amount in gram daily per person; ǂBoth groups combined were also represented as animal products						

Further analysis was made to identify the major types of food items included in the main food groups.

### Cereal

**Figure 2:**
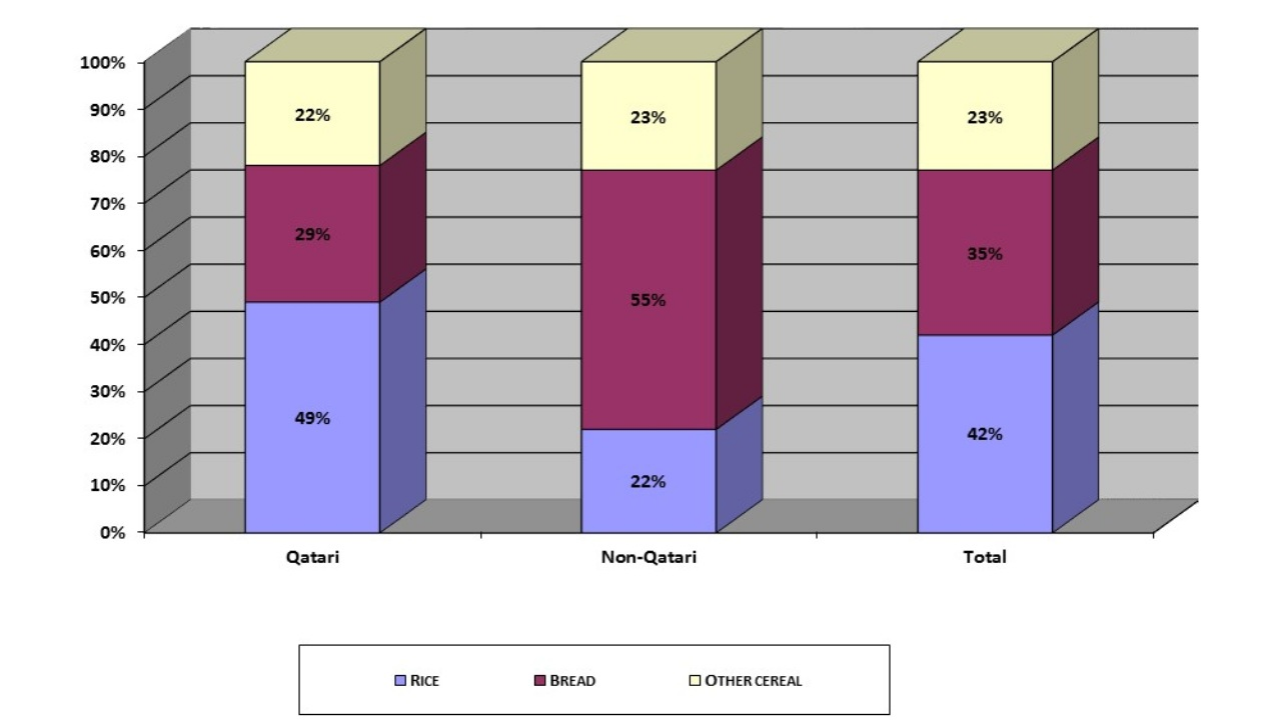
Cereal Patterns by Households in Qatar Total Cereal Amount = 387 g/capita/day (Qatari = 442, Non-Qatari = 279 g/capita/day)

The cereal products were categorized as rice, bread, and other cereal items, 42% of the total cereal products were composed of rice, followed by bread (white and Iranian types) (35%), and rest of the cereals (23%) (Figure 2). Rice was represented as the main item (49%) for the Qatari HHs, while for non-Qatari HHs bread was the main staple cereal (55%). White bread was the main type of bread used by non-Qatari HHs, accounted for 52% of the total cereal products, while for Qatari HHs white bread accounted for 27% of the total cereal products (data not shown here). Different bakery products (cakes, biscuits, oriental sweets, etc.) represented approximately 10% of the cereal group for Qatari and non-Qatari HHs. Other wheat products (macaroni, pasta, wheat flour, etc.) accounted for a percentage in the cereal group among non-Qatari (13%) versus Qatari (12%) (data not shown here).

### Meat

White meat (poultry, fish, and seafood) represented more than half of the meat group (57%) versus red meat products (43%) (Table 2). Lamb was the most commonly consumed food item (30%). A higher proportion of seafood and fish products were purchased among non-Qatari HHs (22%) versus Qatari HHs (18%) (Table 2).

**Table 2:** Pattern of animal meat products purchased in the State of Qatar according to nationality (2012-2013) n = 3723 HHs

	Qatari		Non-Qatari		Total population (Households)	
	Amount* (g)	(%)	Amount*(g)	(%)	Amount*(g)	(%)
Total animal meat group	343.0	100	182.0	100	287.0	100
Lamb	109.8	32	48.5	27	87.5	30
Beef	15.1	4	10.9	6	13.6	5
Beef meat products	23.1	7	13.9	8	20.0	7
Liver	3.2	1	2.7	1	2.9	1
Poultry	128.7	38	65.6	36	107.3	37
Fish	52.5	15	37.7	21	48.0	17
Other sea foods	10.6	3	2.7	1	7.7	3
*(g/capita/day)						

### Milk, dairy, and eggs

Milk, especially in its liquid form, provided about two-thirds of this (Milk, dairy, and eggs) food group/category (65%). Even though the amount of purchased cheese was higher among Qatari HHs (27 g/capita/day) versus non-Qatari HHs (17.5 g/capita/day), it represented just nine percent of the milk, dairy, and eggs category among both nationalities. Around 14% of food items in this category consisted of eggs; among the non-Qatari HHs this figure was slightly higher (15.5%).

### Vegetables and fruit

The group consisted of fresh, frozen, dry, and canned products. The average total amount for this group reached 571 g/capita/day, although the amount for Qatari HHs was higher than non-Qatari HHs by almost 136 g. Vegetables accounted for a slightly higher amount (301 g, 53%) than fruit (270 g, 47%). Qatari HHs purchased equal amounts of fruit (298.1 g, 50%) and vegetables (300.9 g, 50%), while non-Qatari HHs purchased slightly higher amounts of vegetables (258.4 g, 56%) among their nationality group.

### Sugar and sweet

Out of the total for the sugar and sweets group, 73% was made up of sugar (78% Qatari HHs, 69% non-Qatari HHs), four percent honey and jams (eight percent Qatari HHs, seven percent non-Qatari HHs), and 23% chocolate and sweets (14% Qatari HHs, 24% non-Qatari HHs).

### Oils and fats

Qatari HHs obtained more oils and fats in grams than non-Qatari HHs (83 g vs 45 g); however, similar proportions were observed for sub-categories, i.e., vegetable oils (total 90%, Qatari HHs 91%, non-Qatari HHs 89%), butter/ghee (total five percent, Qatari HHs five percent, non-Qatari HHs four percent), and margarine (total five percent, Qatari HHs four percent, non-Qatari HHs seven percent).

### Beverages

About two-thirds (65.5 grams, 62%) of beverages were in carbonated form while the rest (38%) were in the form of juice. Fruit juice accounted for a higher percentage among non-Qatari HHs than Qatari HHs (42% vs 37%).

The data for the following categories: Milk, dairy, and eggs; vegetables and fruit; sugar and sweets; oils and fats; and beverages are not shown here in a table or figure.

### Products under food subsidy system for Qatari nationals

Food subsidy or ration food items consist of rice, milk, oil, and sugar. The products purchased under this system are shown in Table 3. The total amount of subsidized food items reached 428.3 g/capita/day, which accounted for about 20% of total food purchased or acquired by a Qatari HH (2118 g/capita/day) as shown in Table 1. Among food items, subsidized rice and sugar accounted for 90.6% followed by oil (80%). Milk represented only 32.5% of total purchased food amounts (Table 3).

**Table 3:** Subsidized food at Qatari household (2012-2013) n = 1826 HHs

	Total purchased food amount* (g)	Subsidized food items Amount* (g) (%)	
Rice	213.0	193.0	90.6
Milk	186.0	60.4	32.5
Oil	76.0	60.8	80.0
Sugar	119.2	114.1	95.7
Total	594.2	428.3	72.2
*Amount in gram daily per person			

### Nutrient content and nutrient adequacy of food

The daily energy value and macro/micronutrient content of purchased food by nationality are presented in Table 4. Furthermore, Table 5 shows the energy and nutrient adequacy of the diet. On average, acquired food in a Qatari HH contained much higher energy and nutrients than non-Qatari HH. This is mainly due to the subsidized food items (rice, milk, oil, and sugar). Food items listed under the subsidy program provided 1753 kcal/capita/day and accounted for 41% of total daily energy. Fat content in subsidized food items (vegetable oil, milk, and rice) reached 65 g/day and accounted for 38.4% of total dietary fat. The protein contribution from subsidy was 19.3 g/day and accounted for 16.4% of total dietary protein, while fiber from subsidized food provided a very low percentage of dietary fiber (less than 10%).

**Table 4:** Average daily per capita nutrient content of purchased food in the State of Qatar by household (2012-2013)

Household type	Energy (kcal)	Carbohydrate (g)	Protein (g)	Fat (g)	Fiber (g)	Vitamin C (mg)	Vitamin A (ug)	Iron (mg)	Calcium (mg)	Sodium (mg)
Qatari (including subsidized)	4275	569.8	117.7	169.4	7.3	149.6	1273.8	17. 9	877.4	4874.2
Subsidized food*	1753	265	19.3	65	0.6	---	---	---	----	
Non-Qatari	2424	319.6	74.7	94.2	5.2	112.9	883	11.9	604	4636.9
Total surveyed sample	3676	494.8	103.3	142.8	6.4	131.9	1128.8	15.7	793.3	4714.4
* Subsidized food alone (Rice 193 g, oil 60.8 g, sugar 114.1 g, and milk 60.4 g/capita/day)										

According to the age and gender distribution of the members of the surveyed HH (Qatari, non-Qatari, and the total sample), the estimations of the recommended energy and nutrient intake were computed. The food purchased by Qatari HHs provides almost double the estimated requirements for energy, protein, vitamin C, and vitamin A; an adequate amount of iron, and an insufficient amount of calcium (Table 5). For non-Qatari HHs, the energy and nutrient contents of purchased food items were adequate except for iron (69%) and calcium (70%).

**Table 5:** Energy and nutrient adequacy of daily per capita purchased food by household (2012-2013)

Household type		Energy (kcal)	Protein (g)	Vitamin C (mg)	Vitamin A (ug)	Iron (mg)	Calcium (mg)
Qatari	*Estimated requirements*	2157	58.4	41.8	535.8	18.7	1030.6
	*Nutrient content of purchased food*	4275	117.7	149.6	1273.8	17.9	877.4
	*Adequacy %*	198%	202%	358%	242%	96%	85%
Non-Qatari	*Estimated requirements*	2113.7	55.2	40.8	533.1	17.3	862
	*Nutrient content of purchased food*	2424	74.7	112.9	883	11.9	604
	*Adequacy %*	115%	135%	277%	166%	69%	70%
Total surveyed sample	*Estimated requirements*	2143	57.3	41.4	534.96	18.4	976.6
	*Nutrient content of purchased food*	3676	103.3	131.9	1128.8	15.7	793.3
	*Adequacy %*	172%	180%	319%	211%	85%	81%

### Diet quality

Table 6 shows that the protein, fats, and vitamin C had higher levels of nutrient density than the recommended levels as determined by the WHO [16]. It was also found that the carbohydrate and fiber levels had low nutrient density due to dilution of the nutrients throughout the high energy content of purchased food. Vitamin A and calcium were at the lower level of the WHO recommendation except among non-Qataris. Sodium content in purchased food was higher overall among both nationalities (Table 6). The calculations of free sugar levels (not shown in the tables), in terms of dietary type and energy ratios, were greater than the recommendations which is less than 10%. The free sugar levels among Qatari HH (14.9%) and the overall population (13.9%) were high but appropriate among non-Qatari HH (9.5%).

**Table 6:** Nutrient energy density levels of diet in the State of Qatar by household (2012-2013)

Parameters (per 1000 kcal)	Recommended level*	Qatari	Non-Qatari	Total
Carbohydrates (g)	140-190	133.4	131.8	132.6
Proteins (g)	20-25	27.4	30.8	29.2
Fats (g)	16-39	39.4	39.4	39.2
Fiber (g)	8-20	1.7	2.0	2.0
Vitamin C (mg)	25-30	34.9	46.6	32.5
Vitamin A (ug)	350-500	294.3	356.4	302.3
Iron (mg)	3.5-5.5	4.5	5.2	4.6
Calcium (mg)	250-400	203.6	259.2	227.0
Sodium (mg) (as Na Cl)	< 2.5 g	1146.4 = 2.9 g salt NaCl	1818.5 = 4.6 g salt NaCl	1299.8 = 3.3 g salt NaCl
*WHO, 1998 [16]				

## Discussion

The present study is the first attempt to share dietary characteristics of the population in the State of Qatar and provides important information regarding food pattern, nutrition content, requirements, adequacy, and diet quality. Qatar has unique population characteristics, with a population that comprises significant numbers of non-Qatari residents as well as Qatari nationals [8]. This article assesses the similarities and differences in dietary habits among the two groups of nationalities. For instance, it was found that the Qatari HHs purchased about 1.5 times more food in grams per capita per day compared with the non-Qatari HHs; however, the percentages of food groups in the basket were similar for both groups. The composition of the food basket in Qatar was mainly fruit and vegetables, which accounted for approximately 30%, a similar percentage was found for animal products (29%), followed by cereals, which made up 21% of the total basket weight.

It was found that the Qatari diet is high in meat products, sugar, and sodium, and low in calcium and fiber. In addition, the food purchased by the Qatari HHs provided almost double the estimated requirements for energy and protein along with some vitamins. Overall, free sugar from purchased food provides a higher energy percentage than is recommended. Furthermore, the higher daily consumption per capita amounts of sugar, oil, and fats among the Qatari HHs could be due to the fact that these products are readily available through the food subsidy program.

Energy pattern is one of the criteria used to describe diet quality and it identifies the main source of energy in the diet by calculating the percentage contribution of energy supplied from fat, protein, and carbohydrates as well as free sugars to the total dietary energy. Energy pattern for Qatari HHs revealed that the fat/energy ratio was >35%, which is considered high according to the WHO recommendations (15-30%) [13]. The high fat/energy ratio among Qatari HHs could again be a result of the oil that is available through the subsidy system, as evidenced in a recent Qatari study, which showed that the vast majority of respondents (96.4%) used vegetable oil (mainly corn oil) for meal preparation in their HHs [7, 17].

It is recognized that a higher intake of free sugars threatens the nutrient quality of diets by providing significant energy without specific nutrients [15]. Sugar restriction could reduce the risks of unhealthy weight gain [15]. Free sugars calculated from the daily purchased food in Qatar had higher than recommended levels for Qatari HHs. This is also evident from a recent study from Qatar which showed that the consumption of sweets, sugar-sweetened beverages, and fast food was higher among Qatari adults [17].

In Qatar, the total amounts of daily purchased vegetables and fruits were >400 g/capita which is as per accordance with the WHO recommendations [16]. Despite the fact that the amounts of vegetables and fruits were favorable, the fiber content from daily purchased food in the State of Qatar was very low among both Qatari and non-Qatari HHs. Fiber protects against coronary heart disease and has been used in diets to lower blood pressure [18]. Adequate intake may be achieved through eating fruits, vegetables, and wholegrain cereals. Promoting a fiber-rich diet incorporated with healthy diet campaigns would be the best solution toward reduced morbidity and mortality that could be associated with unhealthy dietary habits [17-18]. This is crucial because in 2012 about 17% of adult Qatari nationals were diabetics, 22% had high cholesterol, over 70% were overweight or obese, and a significant relationship existed between obesity and diabetes prevalence [7, 17, 19].

Dietary intake of sodium, from all sources, influences blood pressure levels in populations and hence should be decreased to reduce the risk of coronary heart disease and stroke [20]. Current evidence suggests that an intake of no more than 70 mmol or 1.7 g of sodium per day is beneficial in reducing blood pressure [16]. The study demonstrates that the daily share of sodium from purchased food was above the WHO recommendations. The added daily salt figure per person for the total surveyed sample – Qatari, and non-Qatari household – was about 8 grams, which is considered high. Several studies have investigated the association between dietary patterns and hypertension, consistently showing an increased risk for hypertension with the higher adherence to a “Western” dietary pattern [21]. Given the fact that in 2012 about 33% of Qatari adults were hypertensive, reduction in dietary sodium should be emphasized while implementing new programs [7]. Limiting the intake of dietary sodium to meet these goals should be achieved by restricting daily salt (sodium chloride) intake to less than 5 g per day [15]. To help achieve lower salt consumption, the State of Qatar has adopted the WHO’s salt reduction strategy by notifying certain food manufacturers to reduce salt levels in their products [22].

The consumption of high energy food in excess contributes to increased body weight and various non-communicable disease-related morbidity and mortality. Adoption of policy reform for the food subsidy system will decrease the high amounts of energy consumed. This could be achieved by the introduction of low-fat milk, dry legumes, or other healthier options in the subsidy system. Furthermore, an overall reduction in the amount of sugar via educational campaigns and modification of the subsidy system could also impact on the quality of food. The Qatar Dietary Guidelines, which were developed in response to the results of various local surveys and studies, emphasize the importance of healthy eating such as consuming more legumes, two to four servings of fruits, and three to five servings of vegetables per day [23].

## Conclusions

The HIES provides useful information about types and amounts of the food consumed in Qatar. Food frequency and consumption surveys are essential instruments to assess the dietary situation of a country and provides evidence to support public health policies and programs. The results of the present article would provide useful information to public health professionals, health care workers, and policy makers to support new food consumption-related strategies.

## References

[1] (2017). Food and health data: their use in nutrition policy-making. http://www.who.int/nutrition/publications/policies/isbn9289011254/en/.

[2] (2017). Keynote paper: the use of household expenditure surveys for the assessment of food insecurity. http://www.fao.org/docrep/005/Y4249E/y4249e08.htm.

[3] Danforth E Jr (1985). Diet and obesity. Am J Clin Nutr.

[4] Badran M, Laher I (2011). Obesity in Arabic-speaking countries. Journal Obes.

[5] Badawi A, Arora P, Sadoun E (2012). Prevalence of vitamin D insufficiency in Qatar: a systematic review. J Public Health Res.

[6] Klautzer L, Becker J, Mattke S (2014). The curse of wealth–Middle Eastern countries need to address the rapidly rising burden of diabetes. Int J Health Policy Manag.

[7] (2017). Ministry of Public Health, Qatar STEPwise Report, 2012 Chronic Disease Risk Factor Surveillance. https://d28d0ipak1ih43.cloudfront.net/app/media/download/662.

[8] (2017). Qatar Health Report 2011-2012.

[9] (2017). Ministry of Development Planning and Statistics. Household expenditure and income survey. http://www.mdps.gov.qa/en/statistics/Statistical%20Releases/Social/HouseholdIncomeAndExpenditure/2013/Household_Expenditure_2012_2013_Ar.pdf#search=household%20expenditure.

[10] (2006). Food composition tables for Egypt. National Nutrition Institute.

[11] Musaiger AO (1993). Traditional Foods in the Arabian Gulf Countries, First Edition. http://www.acnut.com/v/images/stories/pdf/publications/traditional_foods_in_the_agc.pdf.

[12] (2017). Human energy requirements. Report of a Joint FAO/WHO/UNU Expert Consultation, Rome. http://www.fao.org/3/a-y5686e.pdf.

[13] (2017). Protein and amino acid requirements in human nutrition. Report of a Joint WHO/FAO/UNU Expert Consultation. http://www.who.int/nutrition/publications/nutrientrequirements/WHO_TRS_935/en/.

[14] (1998). Vitamin and Mineral Requirements in Human Nutrition. http://apps.who.int/iris/bitstream/10665/42716/1/9241546123.pdf.

[15] (2017). Diet, nutrition and the prevention of chronic diseases. Report of a Joint WHO/FAO Expert Consultation. http://apps.who.int/iris/bitstream/10665/42665/1/WHO_TRS_916.pdf.

[16] (2017). Preparation and use of food-based dietary guidelines. Report of a joint FAO/WHO consultation. http://apps.who.int/iris/bitstream/10665/42051/1/WHO_TRS_880.pdf.

[17] Al-Thani MH, Al-Thani AA, Al-Chetachi WF (2015). Dietary and nutritional factors influencing obesity in Qatari adults and the modifying effect of physical activity. J Obes Weight-Loss Medic.

[18] Mendis S, Puska P, Norrving B (2011). Global Atlas on Cardiovascular Disease Prevention and Control. http://apps.who.int/iris/handle/10665/44701.

[19] Al-Thani M, Al-Thani AA, Al-Chetachi W (2017). Situation of diabetes and related factors among Qatari adults: findings from a community based survey. JMIR Diabetes.

[20] Campese VM, Romoff MS, Levitan D (1982). Abnormal relationship between sodium intake and sympathetic nervous system activity in salt-sensitive patients with essential hypertension. Kidney Int.

[21] Bazzano LA, Green T, Harrison TN (2013). Dietary approaches to prevent hypertension. Curr Hypertens Rep.

[22] Alhamad N, Almalt E, Alamir N (2015). An overview of salt intake reduction efforts in the Gulf Cooperation Council countries. Cardiovasc Diagn Ther.

[23] Seed B (2015). Sustainability in the Qatar national dietary guidelines, among the first to incorporate sustainability principles. Public Health Nutr.

